# Diabetic Foot Complications in Saudi Arabia: A Retrospective Study

**DOI:** 10.7759/cureus.53531

**Published:** 2024-02-03

**Authors:** Sherif M Zaki, Dina S El Karsh, Tuleen M Faden, Leen T Almghamsi, Joud O Fathaldin, Omar A Alhazmi

**Affiliations:** 1 Anatomy, Fakeeh College for Medical Sciences, Jeddah, SAU; 2 Family Medicine, Dr. Soliman Fakeeh Hospital, Jeddah, SAU

**Keywords:** saudia arabia, retrospective observational study, chronic diabetic complications, foot complications, diabetes

## Abstract

Review:A common chronic health problem among Saudi Arabians is diabetes mellitus (DM). One of the most serious complications of diabetes is diabetic foot (DMF).

Aim:The objective of this study was to identify the most common complications that develop among patients with DMF. In addition, we conducted a demographic analysis of chronic diabetic complications related to DMF.

Material and methods:The study involved 100 DMF attending the Jeddah clinic of Dr Soliman Fakeeh Hospital. Several chronic complications associated with DMF were reported, including peripheral arterial disease (PAD), coronary artery disease (CAD), retinopathy, nephropathy, and neuropathy. We examined the feet for ulcers, gangrene, amputations, bone deformities, Charcot joints, osteoarthritis, septic arthritis, and osteomyelitis. By using B-mode ultrasound and spectral Doppler imaging, we imaged the posterior tibial and anterior tibial arteries.

Results: People with poorly controlled diabetes mellitus type 2 (T2DM) are more likely to develop diabetic feet. The most common foot complications were foot ulcers (81%), foot amputations (31%), foot gangrene (29%) (29/100), bone deformities (22%) (22/100), ingrown toenails (17%) (17/100), Charcot's foot (10%) (10/100), and calluses (9%) (9/100). The majority of the patients suffered from hypertension and half had anaemia. Diabetic peripheral neuropathy affected about half of the patients, diabetic nephropathy affected one-third, and diabetic retinopathy affected 14%. Approximately a quarter (25/100) of the patients had CAD and less than half had PAD.

There was atherosclerosis in 43% (43/100) of popliteal/infra-popliteal arteries. Twenty-two percent (22/100) of the anterior tibial arteries and 25% (25/100) of the posterior tibial arteries were stenotic or occluded. A biphasic mode was observed in 21% (21/100) of anterior tibial arteries, a monophasic mode in 9% (9/100), and a non-flowing mode in 3% (3/100). Twenty-three percent (23/100) of the posterior tibial arteries displayed biphasic Doppler modes, 5% (5/100) displayed monophasic modes, and 6% (6/100) displayed non-flowing modes.

Conclusion*:* Diabetes foot is common among older males with poorly controlled T2DM. The most common foot complications were amputations, gangrene, foot ulcers, bone deformities, ingrown toenails, Charcot's foot, and calluses. Most DMF patients were anemic and hypertensive. Diabetes-related microvascular complications, such as diabetic peripheral neuropathy, nephropathy, and retinopathy, as well as macrovascular complications, such as coronary artery disease and peripheral arterial disease, were associated with DMF.

## Introduction

Worldwide, 537 million people have diabetes mellitus in 2021. It is expected to reach 643 million in 2030 and 783 million in 2045 [1]. According to the World Health Organization, by 2030 Saudi Arabia will be one of the five countries with the highest prevalence of type 2 diabetes mellitus (T2DM) [1].

The foot, being farthest away from the central nervous system and hemodynamically disadvantageously placed, is the common site of complicated lesions [2]. It is the foot that is most vulnerable to diabetic complications. The diabetic foot is exposed to frequent trauma and relies on sensitive sensory protection, which is often lacking [3]. Diabetic foot (DMF) is one of the most serious complications of diabetes [4]. DMF complications have a prevalence of up to 25% in diabetes patients [5]. Between 20-40% of the funds in diabetes patients are used for foot complications [5]. DMF causes great suffering to the patient and is a major financial burden for the patient's family, healthcare professionals, and society at large [4].

Diabetic foot complications encompass conditions such as diabetic foot ulcers (a break in the skin that occurs below or distal to the malleoli, at least including the epidermis and part of the dermis in a diabetic person), as well as diabetic foot infections (i.e., any infection of the soft tissues or bones of the diabetic foot, including osteomyelitis) [6]. Diabetic foot ulcers (DFUs) are serious complications of diabetes, resulting in significant morbidity and mortality [7]. In total, 19-34% of patients with diabetes develop DFU over their lifetime [8]. Approximately 5% of people with DFU die within the first 12 months, and 42% die within five years [7]. Overall, patients with diabetes are more likely to have an amputation than patients without diabetes [9,10].

DMF was found to be the main cause of lower limb amputation in Saudi Arabia [11]. As a diabetic patient's age and duration of diabetes progresses, his or her risk of ulceration and amputation increases two to fourfold [12]. To make diabetics active partners in their care, the extent of DMF within the Saudi population must be thoroughly disclosed. The objective of this study was to identify the most common complications that develop among patients with DMF. In addition, we conducted a demographic analysis of chronic diabetic complications related to DMF.

## Materials and methods

Hundred DMF patients regularly attending the family medicine clinic at Dr Soliman Fakeeh Hospital in Jeddah, Saudi Arabia, were retrospectively studied. The study ran from March 2022 to August 2023. The participants in the study had to be diagnosed with DFM. Participants under 25 years and those who didn't want to participate were excluded.

The presence of chronic complications such as peripheral vascular disease (PVD), coronary artery disease (CAD), retinopathy, nephropathy, and neuropathy were reported. The diagnosis of PVD was based on either a clinical or physical examination documented in the patient's file. According to our definition, CAD patients were those who had either myocardial infarctions or angina in the past and had either coronary artery bypass grafts or percutaneous transluminal coronary angioplasty procedures in the past. The diagnosis of retinopathy was based on the patient's file. Nephropathy was defined by the albumin excretion in urine. If albumin excretion totals 30-299 g/mg creatinine, it is considered microalbuminuria, whereas if it totals 300, it is considered macroalbuminuria. Neuropathy was considered when the patients were suffering from any form of diabetic neuropathy mainly diabetic polyneuropathy presented by numbness or pain.

Feet were examined for identification of the presence of ulcers, gangrene, and amputation. Foot gangrene was diagnosed when there was tissue death and decay proven by Doppler. Amputation was defined as a minor distal or a major proximal amputation that was related to diabetes. For categorizing and classifying diabetic foot ulcers, Wagner-Meggitt and University of Texas (UT) Staging Systems were used. The Wagner system consists of gradations of superficial ulcers, deep ulcers, abscess osteitis, foot gangrene, and foot gangrene. Based on this system, foot lesions are graded from grade 0 to grade 5. The grade 0 foot has a high risk of developing a lesion, but there is no active lesion, while the grade 5 foot has gangrene throughout. The drawback of this system is that it does not mention ischemia or neuropathy [13]. The UT system is a modification of Wagner's. This system further divides each grade into stages based on the presence of infection, ischemia, or both. In comparison to the Wagner System, this system is somewhat more accurate at predicting outcomes [14,15]. Our study evaluated whether DMF ulcers were neuropathic, ischemic, or multifactorial. Differentiating between neuropathic and ischemic foot ulcers is based on the differences documented in many studies [16,17].

We assessed bone deformities such as pes cavus, hallux valgus, calluses, claw/hammer toes, and fractures of the metatarsal/phalangeal bones. The frequency of Charcot joint, osteoarthritis changes, septic arthritis, and osteomyelitis was also studied. Charcot joint was considered when soft tissues, bones, and joints were inflamed in the presence of neuropathy.

The blood was evaluated for hemoglobin (Hb) (reference level is 14-18 g/dl in males and 12-16 g/dl in females), hemoglobin A1c (HbA1c) (reference level is 7% in diabetes control), C-reactive protein (CRP) (reference level is < 3.0 mg/L) and serum uric acid (reference level is 3.5-7 mg/dL). Furthermore, we assessed total cholesterol levels (normal levels are less than 199 mg/dL, borderline high levels are more than 200 mg/dL), LDL cholesterol levels (normal levels are less than 100 mg/dL, borderline high levels are 100-160 mg/dL, and high levels are over 160 mg/dL), triglycerides (normal levels are less than 150 mg/dL, borderline high levels are 150-199 mg/dL, and high levels are over 200 mg/dL). Additionally, we measured blood urea nitrogen (BUN) levels (reference levels are 8-23 mg/dl), creatinine levels (reference levels are 0.67-1.17 mg/dl), albumin to creatinine ratios (normally less than 30 mg/g), and estimated glomerular filtration rate (eGFR) (reference levels are less than 60 ml/min/1.73 m^2^ bsa).

A radiograph was taken to visualize diabetic foot infections. Using radiography, changes in soft tissues and bone associated with infection could be identified. An ultrasound examination was performed with a Toshiba Xario TUS-X200 (Toshiba Medical System Corporation, Japan) equipped with a 7.5 - 11 MHz transducer. Dorsalis pedis artery sonography was performed supine with the knee bent at 90°, whereas posterior tibial artery sonography was conducted with the limb slightly flexed in the knee and the patient in a lateral position [18]. Initially, the vessels were identified in a transverse plane, and then the probe was rotated perpendicularly to reveal the arteries in a longitudinal plane. Doppler insonation was performed in the longitudinal plane with sample rates of 0.5 mm while maintaining an optimal angle of 60° [18].

The posterior, anterior, and dorsalis pedis arteries were imaged using B-mode ultrasound and spectral Doppler imaging. Using B-mode, the arteries were measured based on their external diameter (from the echogenic outer margin of the near wall to the outer margin of the far wall), plaque presence (defined as areas along the vascular intima causing luminal narrowing that can be homogeneous or heterogeneous, hypoechoic or hyperechoic, or calcium-containing), and the percentage of diameter stenosis that results from these plaques. Spectral Doppler imaging was used to determine the flow spectrum, either triphasic, biphasic, or monophasic [18,19].

Analysis of the data was carried out using the Social Sciences Statistical Package (SPSS), version 26 (IBM Corp., Armonk, NY). The reliability statistics of SPSS were used to determine Cronbach's alpha values. Cronbach's alpha is =0.717, indicating an adequate sample size. Study results indicated that Kaiser-Meyer-Olkin (KMO), a measure of sample adequacy, was 0.8, representing an appropriate sample size. The study analyzed the prevalence of DFUs, gangrene, and amputations, as well as their demographic profiles. There was also a study on the prevalence of other DMF complications. The study also evaluated the association between diabetic foot complications and chronic diabetic complications.

## Results

This study included 100 participants with DFM. The average age of the participants was 60 (10) years. Ninety-eight percent of patients (98/100) had T2DM and two percent (2/100) had Type I diabetes. Seventy-six percent of patients (76/100) had poorly controlled diabetes. Only 3% (3/100) of the participants smoked, 96% (96/100) ex-smoked, and 1% (1/100) didn't smoke.

The prevalence of different ages and sexes

Eight percent of the participants (8/100) were between 25 and 44 years old, 58% (58/100) were 45 to 64 years old, and 34% (34/100) were ≥ 65 years old. Seventy-four percent (74/100) of the participants were male and 26% (26/100) were female.

The prevalence of DMF skin complications

A total of 81% (81/100) of the patients had foot ulcers, 29% (29/100) had gangrene, and 31% (31/100) had amputations. Sixty-seven percent of the patients (67/100) suffered from infections, 32% (32/100) from redness and swelling, 24 % (24/100) from lower limb edema, 17% (27/100) from ingrowing toenails, and 9% (9/100) from calluses (Table 1). Men of advanced age had the highest prevalence of all complications.

**Table 1:** The prevalence of diabetic foot skin complications

	Yes (%)	No (%)
Diabetic foot ulcers	81	19
Infection/abscess	67	33
Redness and swelling	32	68
Gangrene	29	71
Ingrowing toenail	17	83
Callus	9	91
Blister	5	95
Total number of cases	100	

Chronic diabetic complications and their prevalence

Seventy-one percent (71/100) of the patients studied had hypertension, 53% (53/100) had anemia, 49% (49/100) had polyneuropathy, 32% (32/100) had diabetic nephropathy, 14% (14/100) had diabetic retinopathy, 25% (25/100) had CAD, and 43% (43/100) had PAD (Table 2).

Amputees' demographic characteristics

We recorded the provenances of the following data regarding amputation in our study. In 83% (26/31) of cases, diabetes was poorly controlled, hypertension was prevalent in 77% (24/31) of cases, anemia was prevalent in 70% (22/31) of cases, the CRP level was elevated in 45% (14/31) of the cases, the total cholesterol, LDL and triglycerides levels were elevated in 22 % (7/31) of the cases, and hyperuricemia was observed in 9% (3/31). There were also 29% (9/31) of cases with coronary artery disease, 35% (11/31) with diabetic nephropathy, 12% (4/31) with diabetic retinopathy, and 45% (14/31) with diabetic polyneuropathy (Table 2).

Demographic characteristics of gangrenous feet

We recorded the provenance of the following data regarding gangrene in our study. In 83% (25/29) of cases, diabetes was poorly controlled, hypertension was prevalent in 77% (20/29) of cases, anemia was prevalent in 70% (21/29) of cases, the CRP level was elevated in 45% (11/29) of the cases, the total cholesterol, LDL and triglycerides levels were elevated in 22 % (7/31) of the cases, and hyperuricemia was observed in 9% (3/29). There were also 29% (8/29) of cases with coronary artery disease, 35% (13/29) with diabetic nephropathy, 12% (3/29) with diabetic retinopathy, and 45% (13/29) with diabetic polyneuropathy (Table 2).​​​​​​​​​​​​​​

Demographic characteristics of DFU

According to DFU, the following data were recorded in our study. Seventy-seven percent (63/81) of the cases were poorly controlled with diabetes. Hypertension was prevalent in 70% (57/81) of the cases, anemia was prevalent in 58% (47/81) of the cases, the CRP level was elevated in 38% of the cases (31/81) of the cases, the total cholesterol level was elevated in 17% (14/81) of the cases, triglycerides were elevated in 24% (20/81) of the cases, LDL levels were elevated in 23% (19/81) of cases, and hyperuricemia was detected in 7% (6/81). Additionally, 24% (20/81) of the cases suffered from coronary artery disease, 34% (28/81) from diabetic nephropathy, 12% (10/81) from diabetic retinopathy, and 50% (41/81) from diabetic polyneuropathy (Table 2).

**Table 2:** Demographic characteristics of diabetic foot complications Note: DFUs = Diabetic foot ulcers, and LDL = low-density lipoprotein

		DFUs		Amputation		Gangrene		Total
		Non-affected (%)	Affected (%)	Non-affected (%)	Affected (%)	Non-affected (%)	Affected (%)	
Age grouping (years)	25-44 years	4	4	6	2	6	2	8
	45-64 years	9	49	38	20	41	17	58
	≥ 65 years	6	28	25	9	24	10	34
Sex	Male	13	61	46	28	51	23	74
	Female	6	20	23	3	20	6	26
State of control of diabetes	Fair control	6	18	19	5	20	4	24
	Poor control	13	63	50	26	51	25	76
Coronary artery disease	Yes	5	20	16	9	17	8	25
	No	14	61	53	22	54	21	75
Diabetic nephropathy	Yes	4	28	21	11	19	13	32
	No	15	53	48	20	52	16	68
Diabetic retinopathy	Yes	4	10	10	4	11	3	14
	No	15	71	59	27	60	26	86
Diabetic polyneuropathy	Yes	8	41	35	14	36	13	49
	No	11	40	34	17	35	16	51
Charcot foot	Yes	0	10	7	3	6	4	10
	No	19	71	62	28	65	25	90
Hypertension	Yes	14	57	47	24	51	20	71
	No	5	24	22	7	20	9	29
Hyperuricemia	Yes	2	6	5	3	5	3	8
	No	17	75	64	28	66	26	92
Anaemia	Yes	6	47	31	22	32	21	53
	No	13	34	38	9	39	8	47
C-reactive protein	Elevated	0	31	17	14	20	11	31
	Normal	19	50	52	17	51	18	69
Total cholesterol	Elevated	4	14	11	7	11	7	18
	Normal	15	67	58	24	60	22	82
Triglyceride	High	3	5	7	1	6	2	8
	Border-line high	1	15	10	6	10	6	16
	Normal	15	61	52	24	55	21	76
LDL Cholesterol	High	6	19	18	7	17	8	25
	Normal	13	62	51	24	54	21	75
Total		19	81	69	31	71	29	100

The prevalence of DFU (causes and grades) 

Among the patients, 19% (19/100) did not have DFU, whereas 81% (81/100) did. Twenty-eight percent (28/100) of the ulcers were neuropathic, 14% (14/100) were ischemic, and 39% (39/100) were multifactorial. According to the Wagner-Meggitt Classification System, 30% (24/81) of the ulcers were grade 1, 12% (10/81) were grade 2, 20% (16/81) were grade 3, and 38% (31/81) were grade 4.

As per the UT Staging System, 17% (17/100) of the ulcers were Grade 0 - Stage A, 2% (2/100) were Grade 0 - Stage C, 15% (15/100) were Grade I - Stage A, 8% (8/100) were Grade I - Stage B, 1% (1/100) were Grade I - Stage C, 1% (1/100) were Grade I - Stage D, 2% (2/100) were Grade II - Stage A, 6% (6/100) were Grade II - Stage B, 2% (2/100) were Grade II - Stage D, 7% (7/100) were Grade III - Stage A, 19% (19/100) were Grade III - Stage B, 5% (5/100) were Grade III - Stage C and 15% were Grade III - Stage D (Table 3).

**Table 3:** Prevalence of diabetic foot ulcers (cause and grade)

	Diabetic foot ulcers	Frequency
Ulcer cause	No ulcer	19
	Neuropathic ulcer	28
	Ischemic ulcer	14
	Multiple causes-ulcer	39
Ulcer grade according to Wagner-Meggitt Classification System	Grade 0	19
	Grade 1	24
	Grade 2	10
	Grade 3	16
	Grade 4	31
Ulcer grade according to the University of Texas Staging System	Grade 0 - Stage A	17
	Grade 0 - Stage C	2
	Grade I - Stage A	15
	Grade I - Stage B	8
	Grade I - Stage C	1
	Grade I - Stage D	1
	Grade II - Stage A	2
	Grade II - Stage B	6
	Grade II - Stage D	2
	Grade III - Stage A	7
	Grade III - Stage B	19
	Grade III - Stage C	5
	Grade III - Stage D	15
Total number of cases	100	

Lesion prevalence in bones and joints

**Figure 1:**
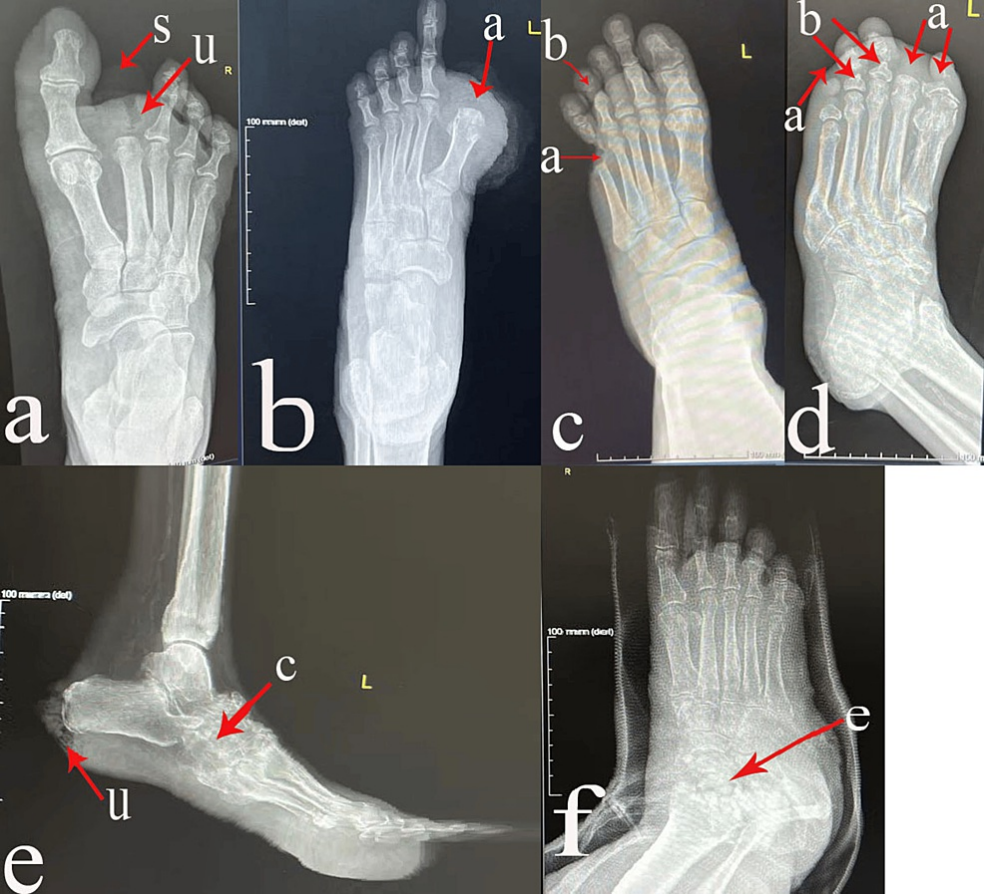
Radiographs of the foot and ankle region of some studied cases a- Forefoot swelling and ulceration (u). Second toe phalangeal bone (s) amputation; b- Hallux amputation (a); c- Deep ulceration accompanied by soft tissue loss and bone loss at the fifth metatarsophalangeal joint (a). There is extensive refraction, erosion, and resorption of the middle and proximal phalanges of the fourth toe (b); d- Hallux, second, and fifth toes were amputated (a). An old fracture of the proximal phalangeal bones of the third and fourth toes (b); e- An ulcer and gas lucencies (u) near the heel region suggest infection or gangrene. The intratarsal and tarsometatarsal joints were affected by erosions, fragmentations, deformities, and Charcot's arthropathy (c); f- The hindfoot is rarefacted, eroded, and fragmented (e).

A total of 22% of patients (22/100) had bone deformities. The distribution of bone deformities was as follows: 11% (11/100) had many deformities, 5% (5/100) had phalangeal deformities, 3% (3/100) had hallux valgus, and the remaining 3% (3/100) had claw/hammer toes. There were 31% (31/100) cases of osteomyelitis, 26% (26/100) of osteoarthritis changes, 10% (10/100) of Charcot's foot, and 2% (2/100) of septic arthritis cases (Table 4, Figure 1).

**Table 4:** Lesion prevalence in bones and joints

Bone deformities	Type	Frequency
	No deformity	78
	Metatarsal/phalangeal bone fracture	5
	Hallux valgus	3
	Claw/Hammer toe	3
	Many deformities	11
Joint lesions	Yes (%)	No (%)
Charcot joint	10	90
Osteoarthritic changes	26	74
Septic arthritis	2	98
Osteomyelitis	31	69
Total number of cases	100	

Analyses of laboratory investigations

There were 6% (6/100), 28% (28/100), 14% (14/100), and 23% (23/100) patients with elevated BUN, elevated creatinine, and elevated albumin/creatinine ratios, respectively. The total cholesterol of 18% (18/100), the LDL cholesterol of 24% (24/100), and the triglycerides of 1% (1/100) were borderline high, while 16 % (16/100) had borderline high triglycerides, and 8% (8/100) had high triglycerides. Adding, there were 6% (6/100), 28% (28/100), 14% (14/100), and 23% (23/100) patients with elevated BUN, creatinine, albumin/creatinine ratios, and decreased eGFR, respectively (Table 5).​​​​​​​

**Table 5:** Lab investigations of the studied patients Note: Hb = hemoglobin, HbA1c = hemoglobin A1c, CRP =C-reactive protein, BUN = blood urea nitrogen, eGFR = estimated glomerular filtration rate and LDL = low-density lipoprotein

	Affected (%)	Normal (%)
Hb	53 (Anemic)	47
HbA1c	76 (Poor control of diabetes)	24 (fair control of diabetes)
CRP	31 (increased)	69
BUN	6 (increased)	94
Creatinine (enzymatic)	28 (increased)	70
	2 (decreased)	
eGFR	23 (deceased)	77
Albumin/ creatinine ratio	14 (microalbuminuria)	86
Uric acid	17 (elevated)	83 (normal)
Total cholesterol	18 (borderline high)	82 (normal)
LDL cholesterol	24 (borderline high)	75 (normal)
	One high	
Triglyceride	16 (borderline high)	76 (normal)
	8 high	

The infra-popliteal arteries' demographics

**Figure 2:**
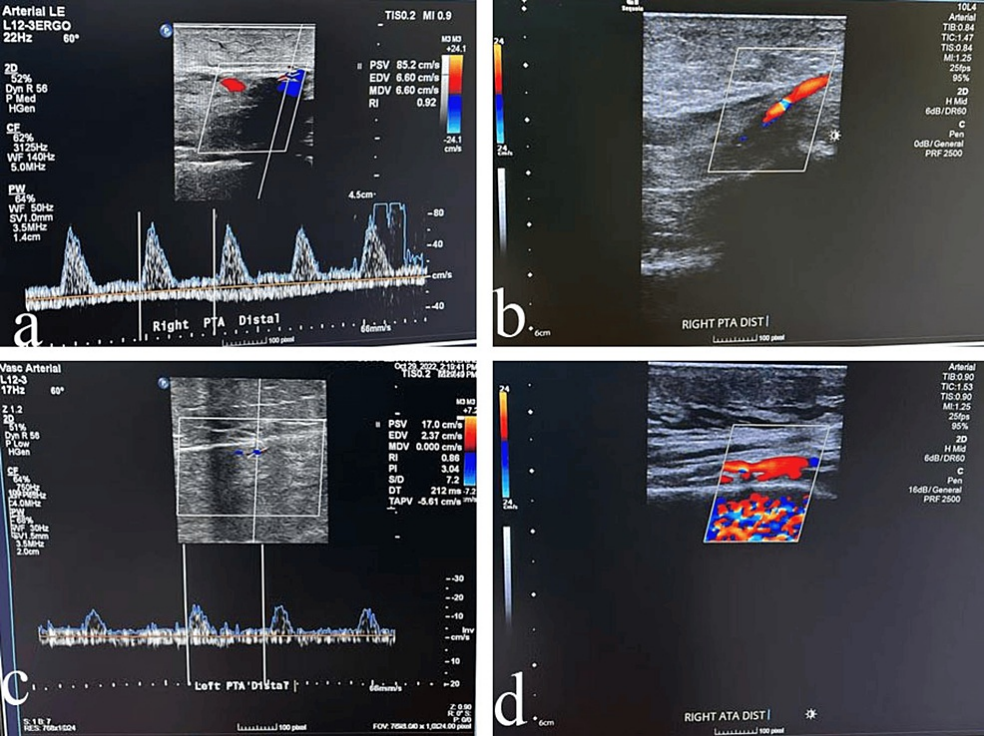
Triplex Doppler sonogram of some studied cases a- The right posterior tibial artery has been attenuated by atherosclerosis with triphasic to biphasic waveforms and no definite segment of occlusion; b- The right posterior tibial artery is attenuated by atherosclerosis without definite obstruction; c- The left posterior tibial artery is patent with segmental attenuation. There are triphasic to biphasic waveforms; d- The left anterior tibial artery is attenuated by atherosclerosis without definite obstruction.

Forty-three percent (43/100) of the infra-popliteal arteries had intimal plaques. About 75% (75/100) of the posterior tibial arteries were patent, 13% (13/100) were stenotic, and 12% (12/100) were occluded. Dorsalis pedis and anterior tibial arteries were patent in 76% (76/100) of the cases, stenotic in 13% (13/100), and occluded in 9% (9/100). Sixty-six percent (66/100) of the posterior tibial arteries had a triphasic Doppler mode, 23% (23/100) had a biphasic mode, 5% (5/100) had a monophasic mode, and 6% (6/100) had no flow. Sixty-seven percent (67/100) of the anterior tibial and dorsalis pedis arteries had triphasic Doppler modes, 21% (21/100) had biphasic modes, 9% (9/100) had monophasic modes, and 3% (3/100) had non-flowing modes (Table 6, Figure 2). DVT occurred in two percent of the cases (2/100).​​​​​​​

**Table 6:** Doppler mode of the infra-popliteal arteries

	Doppler mode	Frequency
Intimal plaques of the infra-popliteal arteries	No plaque	54
	Hypoechoic	2
	Hyperechoic	1
	Calcified	43
Diameter stenosis of the posterior tibial arteries	Stenotic	13
	Patent	75
	Occluded	12
Diameter stenosis of the anterior tibial and dorsalis pedis arteries	Stenotic	13
	Patent	78
	Occluded	9
Spectral pattern of the posterior tibial arteries.	Triphasic (Normal)	66
	Biphasic	23
	Monophasic	5
	No flow	6
Spectral pattern of the anterior tibial and dorsalis pedis arteries	Triphasic (Normal)	67
	Biphasic	21
	Monophasic	9
	No flow	3

## Discussion

In the 45-64 years age group, 49% (49/100) of the cases have DFU, 20% (20/100) have amputated feet, and 10% (10/100) have gangrenous feet. Over the age of 65 years, 28% (10/100) of the cases have DFU, 17% (17/100) of cases have gangrenous feet and 9% (9/100) have amputated feet. Among the older cases, 28% (28/100) have DFU, 17% (17/100) have gangrene and 9% (9/100) have amputated feet. Susceptibility to complications from diabetic foot is strongly influenced by age [20]. The mean age plays a significant role in the occurrence of foot ulcers, gangrene, and amputations [20]. The mean age plays a significant role in the occurrence of foot ulcers, gangrene, and amputations [21,22].

According to our study, diabetic foot cases are more prevalent in males (74%) (74/100). It is consistent with data from other studies showing that males have a higher incidence and prevalence of diabetes and diabetes-related complications (ulcers, gangrene, amputations) than females [23-25]. There are several reasons why males are more likely to suffer from diabetes and its complications. Trauma and inadequate footwear are more common among men [26,27]. Trauma and inadequate footwear are more common among men [26,27]. Additionally, women have a greater awareness of self-care and a positive body-care mood and are more involved in preventative and self-care activities [28].

Most of the studied cases (76%) (76/100) had poorly controlled diabetes. There was a poorly controlled diabetes problem in 77% (63/81) of those with DFU, 86% (25/29) of those with gangrenous feet, and 83% (26/31) of those with amputated feet. As reported previously, patients with poor glycaemic control are more likely to develop DFUs and are more likely to develop diabetic foot lesions [17,29].

Among the studied cases, 53% (58/100) were anaemic. The prevalence of anemia was 58 percent in patients with DFU (47/81), 70 percent in amputees (22/31), and 72% in gangrenous patients (21/29). For wound healing to take place, oxygen is crucial [30]. Oxygen prevents infection, facilitates the differentiation and reepithelialization of keratinocytes, promotes fibroblast proliferation and collagen synthesis, and accelerates wound healing [30]. Hemoglobin (Hb) is the major carrier of oxygen in the blood [30]. Consequently, anemia can impede ulcer healing by reducing oxygen delivery at the site of the ulcer [30]. Wounds heal slowly, and amputation is more likely when anemia is present [31].

Seventy-one percent (71/100) of the DMF in our study had hypertension (BP >130/80 mmHg). The increased blood pressure in diabetics is due to the increased viscosity of the blood, resulting in a decreased flow of blood, which leads to vascular deficiency [32]. Seventy-one percent (57/81) of the DFU patients in our study were hypertensive. The endothelium can become damaged or swollen when pressure exceeds 130/80 mmHg, leading to ulcers [32].

Amputation, foot ulcers, and gangrene were associated with lower cholesterol, LDL, and triglyceride levels. This may be because most of the cases were being treated with lipid-lowering agents at the time of analysis [25]. In T2DM, dyslipidemia occurs when the cells cannot metabolize glucose, resulting in the mobilization of fats, leading to high blood fatty acid levels [33]. 

Among our participants, 49% (49/100) had diabetic polyneuropathy, 32% (32/100) had diabetic nephropathy, and 14%(14/100) had diabetic retinopathy. Diabetic peripheral neuropathy, retinopathy, and nephropathy are microvascular complications of late diabetes [34]. Diabetes nephropathy may cause foot lesions and/or delay healing [35], while diabetic retinopathy may increase the chances of foot trauma [36].

A quarter of our participants suffered from Coronary artery disease (CAD). Coronary artery disease is one of the macrovascular complications of late diabetes. Atherosclerosis is the primary pathological process involved in macrovascular disease, which results in the narrowing of arterial walls throughout the body [37].

Forty-three percent (14/100) of popliteal/infra-popliteal arteries in our case had atherosclerotic changes. Atherosclerosis is one of the most feared complications of diabetes mellitus [38]. An important manifestation of systemic atherosclerosis is peripheral arterial disease (PAD), which is characterized by occlusive changes in the lower limb arteries [39]. PAD is another macrovascular complication of late diabetes [37]. Atherosclerotic disorders affecting the extra-coronary circulation are encompassed by the term PAD [40]. Lower extremity peripheral arterial disease (LEPAD) is PAD affecting the lower limb arteries [40]. Atherosclerosis occurs due to chronic inflammation and injury of the arterial wall [41]. Oxidized lipids from LDL particles accumulate in the endothelial wall of the arteries as a result of endothelial injury and inflammation [41].

Approximately 22% (22/100) of the anterior tibial/dorsalis pedis and 25% (25/100) of posterior tibial arteries were stenotic or occluded. Twenty-one percent (21/100) of the anterior tibial and dorsalis pedis arteries had biphasic Doppler modes, 9% (9/100) had monophasic modes, and 3% (3/100) had non-flowing modes. Twenty-three percent (23/100) of the posterior tibial arteries had a biphasic Doppler mode, 5% (5/100) had a monophasic mode, and 6% (6/100) had no flow. The waveforms of each segment of the vascular tree can be used to locate any occlusions or stenosis [40]. Triphasic waveforms correspond to the three phases of a heartbeat (systole, diastole, elastic recoil) [40]. A biphasic waveform indicates mild to moderate disease, while a monophasic waveform indicates significant disease [40]. In previous studies, the prevalence of LEPAD was reported as 14% in Sudanese diabetic patients, 25% in India, 28% in South Korea, 30% in Poland, 30% in Ethiopia, 33% in Nigeria, 54% in Germany, and 65% in Pakistan [40,42-47].

We observed ulcers, skin infections, redness and swelling, gangrenous feet, edema of the lower limbs, blisters, and skin infections as the most common complications in our study. Such findings are in line with other data sources indicating foot ulcerations and wounds, amputations, and infections are common complications among diabetics [48]. The presence of skin changes in the diabetic foot may indicate that amputation is imminent [49]. In our study, 31% of patients had an amputation. Skin infections accounted for 67% (67/100) of the cases, redness and swelling 32% (32/100), and lower limb edema 24% (24/100). There are several possible causes of foot infection, including neuropathy and ischemia [50]. A loss of sweat gland function can also lead to dry, cracked skin that becomes infected [51]. Erythema, edema, purulence, increased drainage, and malodor are characteristics of active foot infections [48].

We found that 17% (17/100) of participants had ingrown toenails. Ingrown nails with concomitant infection and infected skin ulcers are important risk factors for amputations [49]. Nine percent (9/100) of the participants in our study had calluses. Callus development is caused by a variety of factors, peripheral neuropathy being the most important [52]. As a result of motor neuropathy, the feet may develop deformities, and sensory neuropathy will cause a lack of sensation, leading to persistent abnormal pressure on the feet [52]. In response to it, skin cells increase keratinization and form calluses, which lead to foot ulcers [52]. A callus significantly increases the risk of developing a foot ulcer [52].

Ten percent of the studied patients (10/100) had Charcot's foot. The term Charcot's foot refers to bone and joint destruction in the neuropathy foot. Charcot arthropathy is characterized by dislocation, debris, disorganization, and changes in bone density [53]. In our study, a 10% (10/100) prevalence of Charcot's foot is associated with foot ulcers, a 3% (3/100) prevalence with amputated feet, and a 4% (4/100) prevalence with gangrenous feet. Likewise, other studies have shown sevenfold increases in the relative risk of amputation and fourfold increases in the relative risk of foot ulceration among Charcot joint patients [54,55].

A total of 22% (22/100) of the study's patients had foot bone deformities. The prevalence of phalangeal deformities was 5% (5/100), hallux valgus was 3% (3/100), and claws/hammer toes were 3% (3/100). Metatarsal head deformities and clawing of the toes are common consequences of motor neuropathy [51]. Typically, motor neuropathy results from the wasting of the intrinsic muscles of the feet, causing clawing of the toes and changes in midfoot architecture, resulting in pressure redistribution over the metatarsals [56]. In addition, hammer toes can also be caused by nerve degeneration of the lumbricals [56].

Two main risk factors that contribute to the development of foot ulcers in diabetics are peripheral neuropathy (sensory, motor, and autonomic) and PVD [57,58]. It is also important to note that trauma plays a significant role in ulcer development [58]. A total of 28% of the ulcers in our study were neuropathic, 14% were ischemic, and 39% were multifactorial.

To study the DFU, we used two classification systems, the Wagner-Meggitt System and the UT Staging System. Wagner-Meggitt is limited in its coverage of ischemia and neuropathy [13]. UT's system is somewhat more accurate at predicting diabetic foot wound outcomes than Wagner's [14,15]. Diabetic foot ulcers are commonly classified and categorized using the UT system [14,15]. Based on this system, one-fifth of the ulcers were Grade 0 - Stage A, 2% were Grade 0 - Stage C, 15% were Grade I - Stage A, 8% were Grade I - Stage B, 1% were Grade I - Stage C, 1% were Grade I - Stage D, 2% were Grade II - Stage A, 6% were Grade II - Stage B, 2% were Grade II - Stage D, 7% were Grade III - Stage A, 19% were Grade III - Stage B, 5% were Grade III - Stage C, and 15% were Grade III - Stage D.

## Conclusions

Diabetes foot is common among older males with poorly controlled T2DM. The most common foot complications were amputations, gangrene, foot ulcers, bone deformities, ingrown toenails, Charcot's foot, and calluses. Most DMF patients were anemic and hypertensive. Diabetes-related microvascular complications, such as diabetic peripheral neuropathy, nephropathy, and retinopathy, as well as macrovascular complications, such as coronary artery disease and peripheral arterial disease, were associated with DMF.

## References

[1] Tönnies T, Rathmann W, Hoyer A, Brinks R, Kuss O (2021). Quantifying the underestimation of projected global diabetes prevalence by the International Diabetes Federation (IDF) Diabetes Atlas. BMJ Open Diabetes Res Care.

[2] Gottrup F (2004). A specialized wound-healing center concept: importance of a multidisciplinary department structure and surgical treatment facilities in the treatment of chronic wounds. Am J Surg.

[3] Sinwar PD (2015). The diabetic foot management-recent advance. Int J Surg.

[4] Schaper NC, van Netten JJ, Apelqvist J, Bus SA, Hinchliffe RJ, Lipsky BA (2020). Practical guidelines on the prevention and management of diabetic foot disease (IWGDF 2019 update). Diabetes Metab Res Rev.

[5] Lepäntalo M, Apelqvist J, Setacci C (2011). Chapter V: diabetic foot. Eur J Vasc Endovasc Surg.

[6] van Netten JJ, Bus SA, Apelqvist J (2020). Definitions and criteria for diabetic foot disease. Diabetes Metab Res Rev.

[7] Everett E, Mathioudakis N (2018). Update on management of diabetic foot ulcers. Ann N Y Acad Sci.

[8] Armstrong DG, Boulton AJ, Bus SA (2017). Diabetic foot ulcers and their recurrence. N Engl J Med.

[9] Reiber GE, Boyko EJ, Smith DG (1995). Lower extremity foot ulcers and amputations in diabetes. Diabetes in America.

[10] Wukich DK, Raspovic KM, Suder NC (2018). Patients with diabetic foot disease fear major lower-extremity amputation more than death. Foot Ankle Spec.

[11] Al-Khaldi YM (2008). Foot care among male diabetics in family practice center, abha, saudi arabia. J Family Community Med.

[12] Katsilambros N, Dounis E, Makrilakis K, Tentolouris N, Tsapogas P (2010). Atlas of the diabetic foot. https://books.google.com.sa/books/about/Atlas_of_the_Diabetic_Foot.html?id=mKj1MLUnlekC&redir_esc=y.

[13] Jain AKC (2012). A new classification of diabetic foot complications: a simple and effective teaching tool. J Diab Foot Comp.

[14] Armstrong DG, Lavery LA, Harkless LB (1998). Validation of a diabetic wound classification system. The contribution of depth, infection, and ischemia to risk of amputation. Diabetes Care.

[15] Armstrong DG, Lavery LA, Harkless LB (1996). Treatment-based classification system for assessment and care of diabetic feet. J Am Podiatr Med Assoc.

[16] Oyibo SO, Jude EB, Voyatzoglou D, Boulton AM (2002). Clinical characteristics of patients with diabetic foot problems: changing patterns of foot ulcer presentation. Practical Diabetes International.

[17] Singh N, Armstrong DG, Lipsky BA (2005). Preventing foot ulcers in patients with diabetes. JAMA.

[18] Leoniuk J, Lukasiewicz A, Szorc M, Sackiewicz I, Janica J, Lebkowska U (2014). Doppler ultrasound detection of preclinical changes in foot arteries in early stage of type 2 diabetes. Pol J Radiol.

[19] Myers K, Clough A (2004). Making sense of vascular ultrasound: a hands-on guide. https://www.taylorfrancis.com/books/mono/10.1201/b13409/making-sense-vascular-ultrasound-kenneth-myers-amy-clough.

[20] Stancu B, Ilyés T, Farcas M, Coman HF, Chiș BA, Andercou OA (2022). Diabetic foot complications: a retrospective cohort study. Int J Environ Res Public Health.

[21] Shahi SK, Kumar A, Kumar S, Singh SK, Gupta SK, Singh T (2012). Prevalence of diabetic foot ulcer and associated risk factors in diabetic patients from North India. The Journal of Diabetic Foot Complications.

[22] Cerqueira LO, Duarte EG, Barros AL, Cerqueira JR, de Araújo WJ (2020). WIfI classification: the Society for Vascular Surgery lower extremity threatened limb classification system, a literature review. J Vasc Bras.

[23] Al-Mahroos F, Al-Roomi K (2007). Diabetic neuropathy, foot ulceration, peripheral vascular disease and potential risk factors among patients with diabetes in Bahrain: a nationwide primary care diabetes clinic-based study. Ann Saudi Med.

[24] Bruun C, Siersma V, Guassora AD, Holstein P, de Fine Olivarius N (2013). Amputations and foot ulcers in patients newly diagnosed with type 2 diabetes mellitus and observed for 19 years. The role of age, gender and co-morbidity. Diabet Med.

[25] Al-Rubeaan K, Al Derwish M, Ouizi S, Youssef AM, Subhani SN, Ibrahim HM, Alamri BN (2015). Diabetic foot complications and their risk factors from a large retrospective cohort study. PLoS One.

[26] Ansari S, Akhdar F, Mandoorah M, Moutaery K (2000). Causes and effects of road traffic accidents in Saudi Arabia. Public health.

[27] Al-Wahbi AM (2006). The diabetic foot. In the Arab world. Saudi Med J.

[28] Hjelm K, Nyberg P, Apelqvist J (2002). Gender influences beliefs about health and illness in diabetic subjects with severe foot lesions. J Adv Nurs.

[29] Deshpande AD, Harris-Hayes M, Schootman M (2008). Epidemiology of diabetes and diabetes-related complications. Phys Ther.

[30] Kumar R, Singh SK, Agrawal NK, Kumar U, Kumar S, C S, Bishnoi A (2023). The prevalence of anemia in hospitalized patients with diabetic foot ulcer (DFU) and the relationship between the severity of anemia and the severity of DFU. Cureus.

[31] Yammine K, Hayek F, Assi C (2021). Is there an association between anemia and diabetic foot ulcers? A systematic review and meta-analysis. Wound Repair Regen.

[32] Prabowo E (2018). Risk factors analysis of diabetic foot ulcers among individual with diabetes mellitus. UNEJ E-Proceeding.

[33] Marieb EN, Hoehn K (2007). Human anatomy & physiology. Human anatomy & physiology. Pearson education.

[34] Frykberg RG, Zgonis T, Armstrong DG (2006). Diabetic foot disorders: a clinical practice guideline (2006 revision). The journal of foot and ankle surgery.

[35] Deery HG 2nd, Sangeorzan JA (2001). Saving the diabetic foot with special reference to the patient with chronic renal failure. Infect Dis Clin North Am.

[36] Maurer MS, Burcham J, Cheng H (2005). Diabetes mellitus is associated with an increased risk of falls in elderly residents of a long-term care facility. J Gerontol A Biol Sci Med Sci.

[37] Chawla A, Chawla R, Jaggi S (2016). Microvasular and macrovascular complications in diabetes mellitus: distinct or continuum?. Indian J Endocrinol Metab.

[38] Hiatt WR (2001). Medical treatment of peripheral arterial disease and claudication. N Engl J Med.

[39] Shaheen R, Sohail S (2010). A doppler-based evaluation of peripheral lower limb arterial insufficiency in diabetes mellitus. Journal of the College of Physicians and Surgeons--Pakistan: JCPSP.

[40] Oduola-Owoo LT, Adeyomoye AA, Olowoyeye OA, Odeniyi IA, Idowu BM, Oduola-Owoo BB, Aderibigbe AS (2022). Comparative Doppler ultrasound findings of foot arteries in patients with type 2 diabetes mellitus and normoglycaemic patients. J West Afr Coll Surg.

[41] Boyle PJ (2007). Diabetes mellitus and macrovascular disease: mechanisms and mediators. Am J Med.

[42] Hur KY, Jun JE, Choi YJ (2018). Color doppler ultrasonography is a useful tool for diagnosis of peripheral artery disease in type 2 diabetes mellitus patients with ankle-brachial index 0.91 to 1.40. Diabetes Metab J.

[43] Agboghoroma OF, Akemokwe FM, Puepet FH (2020). Peripheral arterial disease and its correlates in patients with type 2 diabetes mellitus in a teaching hospital in northern Nigeria: a cross-sectional study. BMC Cardiovasc Disord.

[44] Janssen A (2005). Pulsatility index is better than ankle-brachial doppler index for non-invasive detection of critical limb ischaemia in diabetes. Vasa.

[45] Akalu Y, Birhan A (2020). Peripheral arterial disease and its associated factors among type 2 diabetes mellitus patients at Debre Tabor general hospital, Northwest Ethiopia. J Diabetes Res.

[46] Ali RI, Suliman AG, Abdelrahim A, Gameraddin M (2022). A triplex ultrasound evaluation of preclinical changes in type 2 diabetes in foot arteries. Cureus.

[47] Shaheen R, Sohail S (2010). A Doppler-based evaluation of peripheral lower limb arterial insufficiency in diabetes mellitus. J Coll Physicians Surg Pak.

[48] Kim PJ, Steinberg JS (2013). Complications of the diabetic foot. Endocrinol Metab Clin North Am.

[49] Itin PH (1999). The diabetic foot--the view of the dermatologist (Article in German). Praxis (Bern 1994).

[50] Bandyk DF (2018). The diabetic foot: pathophysiology, evaluation, and treatment. Semin Vasc Surg.

[51] Ahmad J (2016). The diabetic foot. Diabetes Metab Syndr.

[52] Arosi I, Hiner G, Rajbhandari S (2016). Pathogenesis and treatment of callus in the diabetic foot. Curr Diabetes Rev.

[53] Ledermann H, Morrison W, Schweitzer M (2012). Imaging of the foot & ankle: techniques and applications. https://www.abebooks.com/first-edition/Imaging-Foot-Ankle-Techniques-Applications-Medical/31059721983/bd.

[54] Sohn MW, Stuck RM, Pinzur M, Lee TA, Budiman-Mak E (2010). Lower-extremity amputation risk after Charcot arthropathy and diabetic foot ulcer. Diabetes Care.

[55] Boyko EJ, Ahroni JH, Stensel V, Forsberg RC, Davignon DR, Smith DG (1999). A prospective study of risk factors for diabetic foot ulcer. The Seattle Diabetic Foot Study. Diabetes Care.

[56] Reardon R, Simring D, Kim B, Mortensen J, Williams D, Leslie A (2020). The diabetic foot ulcer. Aust J Gen Pract.

[57] Nouira S, Ach T, Bellazreg F, Ben Abdelkrim A (2023). Predictive factors for lower limb amputation in type 2 diabetics. Cureus.

[58] Bus SA, van Deursen RW, Armstrong DG, Lewis JE, Caravaggi CF, Cavanagh PR (2016). Footwear and offloading interventions to prevent and heal foot ulcers and reduce plantar pressure in patients with diabetes: a systematic review. Diabetes Metab Res Rev.

